# Prognostic Role of Anemia in COVID-19 Patients: A Meta-Analysis

**DOI:** 10.3390/idr13040085

**Published:** 2021-10-31

**Authors:** Marco Zuin, Gianluca Rigatelli, Laura Quadretti, Luisella Fogato, Giovanni Zuliani, Loris Roncon

**Affiliations:** 1 Department of Translational Medicine, Section of Internal and Cardio Respiratory Medicine, University of Ferrara, 44124 Ferrara, Italy; giovanni.zuliani@unife.it; 2 Department of Cardiology, Rovigo General Hospital, 45100 Rovigo, Italy; jackyheart@libero.it (G.R.); roncon.loris@gmail.com (L.R.); 3 Department of Medicine, Casa di Cura Porto Viro, 45014 Rovigo, Italy; quadrettilaura@gmail.com; 4 Department of General Surgery, Santa Maria degli Angeli Hospital, 06081 Adria, Italy; fogato.luisella@gmail.com

**Keywords:** anemia, COVID-19, prevalence, mortality

## Abstract

Introduction. The prevalence and prognostic implications of anemia in patients infected by the SARS-CoV-2 remains unclear. We performed a systematic review and meta-analysis to assess the prevalence and mortality risk in COVID-19 patients with anemia. Methods. Preferred Reporting Items for Systematic Reviews and Meta-Analyses guidelines were followed in abstracting data and assessing validity. We searched MEDLINE and Scopus to locate all the articles published up to 1 September 2021, reporting data on the adjusted OR (aOR) for mortality among COVID-19 patients with anemia. The pooled prevalence of anemia among COVID-19 patients was calculated using a random effects model and presenting the related 95% confidence interval (CI), while the mortality risk was estimated using the Mantel-Haenszel random effects models with odds ratio (aOR) and related 95% CI. Statistical heterogeneity was measured using the Higgins I^2^ statistic. Results. Five studies, enrolling 9.623 COVID-19 patients [3.707 males (38.5%)], met the inclusion criteria and were included in the final analysis. The pooled prevalence of anemia was 25.6% of cases (95% CI: 8.3–56.5%), with high heterogeneity (I^2^ = 98.9%). Meta-regression showed that the anemia prevalence was influenced by a direct correlation with age (*p* = 0.007) and chronic kidney disease (*p* = 0.004) as moderating variables. Conversely, an inverse relationship was observed with male gender (*p* < 0.0001). Anemia was significantly associated with higher risk of short-term mortality (aOR: 1.69, 95% CI: 1.28–2.24, *p* < 0.001), with low heterogeneity (I^2^ = 0%). Conclusions. Anemia represents a major comorbidity in about 25% of COVID-19 patients and it is associated with about 70% higher risk of short-term mortality.

## 1. Introduction

Over the last two years, several analyses have demonstrated that the prognosis of patients with COVID-19 infection is closely related to the burden of associated comorbidities, such as arterial hypertension (HT), atrial arrhythmias, diabetes mellitus (DM) and more generally to cardiovascular disease (CVDs) [1,2,3,4,5]. However, few investigations, often achieving conflicting results, have focused their attention on the association between anemia in COVID-19 patients and the related risk of short-term mortality [6,7]. Conversely, recent studies have demonstrated that pre-existing anemia represents a risk factor for severe COVID-19 infection [8,9]. As known, anemia remains a global health concern, representing a significant risk factor for hospitalization and/or mortality, especially in those patients infected by the SARS-CoV-2 virus who present other comorbidities associated with a poor prognosis [10,11,12]. Indeed, anemia can further reduce oxygen delivery to peripheral tissue in COVID-19 patients who have an increased oxygen demand due the interstitial pneumonia. Therefore, in the present study we estimated the pooled prevalence and the influence of anemia on short-term mortality, defined as in-hospital mortality, in COVID-19 patients by a systematic review and meta-analysis of the available data.

## 2. Materials and Methods

### 2.1. Data Sources and Searches

The study was performed in accordance with the Preferred Report Items for Systematic Reviews and Meta-analyses (PRISMA) guidelines (Supplementary File S1) [13]. PubMed and Scopus databases were systematically searched for articles, published in English language, from inception through 1 September 2021, with the following Medical Subject Heading (MESH) terms: “COVID-19” OR “SARS-CoV-2” AND “Anemia”. In addition, references from the included studies were screened to potentially identify other investigations meeting the inclusion criteria. 

### 2.2. Study Selection

Specifically, inclusion criteria were: (i) studies enrolling subjects with a confirmed diagnosis of COVID-19; (ii) studies providing data on the prevalence of anemia among patients enrolled and (iii) adjusted odds ratios (aORs) estimating the short-term mortality risk among COVID-19 patients with anemia. Conversely, case reports, review articles, abstracts, editorials/letters, and case series with less than 10 participants were excluded. Each included article was independently evaluated by two reviewers (MZ, GR); in case of discrepancies a third author was involved (LF), and final consensus was achieved through discussion. 

### 2.3. Data Extraction and Quality Assessment 

Data were independently extracted by two reviewers (MZ, GR and LQ) using a standardized protocol. Disagreements, if any, were resolved by discussion. For this meta-analysis, the following data elements were extracted: sample size, mean age, number of non-survivors (NS), male gender, prevalence of anemia, major comorbidities such as HT, DM, cancer, heart failure (HF), coronary artery disease (CAD), chronic obstructive pulmonary disease (COPD) and chronic kidney disease (CKD) as well as the aOR for short-term mortality in COVID-19 patients with anemia. The quality of included studies was graded using the Newcastle–Ottawa quality assessment scale (NOS) [14]. 

### 2.4. Outcomes

The prevalence of anemia in COVID-19 patients was chosen as the primary outcome, while its associated mortality risk was selected as the secondary outcome.

### 2.5. Data Synthesis and Analysis

Continuous variables were expressed as median while categorical variables were expressed as counts and percentages. The cumulative prevalence of anemia (*n*/N), defined as the ratio between patients with anemia (*n*) and the number of patients enrolled in each study (N), were pooled using a random effects model and presented with the corresponding 95% confidence interval (CI). Moreover, to estimate the mortality risk, data were pooled using the Mantel–Haenszel random effects model with odds ratio (OR) as the effect measure with 95% CI. Heterogeneity among studies was assessed using the Higgins and Thomson I^2^ statistic [15]. The presence of potential publication bias was verified by visual inspection of the funnel plot. Due to the low number of the included studies (<10), small-study bias was not examined as our analysis was underpowered to detect such bias. A predefined sensitivity analysis (leave-one-out analysis) was performed removing 1 study at the time, to evaluate the stability of our results regarding the mortality risk. To further appraise the impact of potential baseline confounders in the estimation of the polled prevalence of anemia, a meta-regression analysis using age, gender, HT, DM and CKD as moderator variables was performed. All meta-analyses were conducted using Comprehensive Meta-Analysis software, version 3 (Biostat, Dallas, TX, USA).

## 3. Results

### 3.1. Search Results 

**Figure 1:**
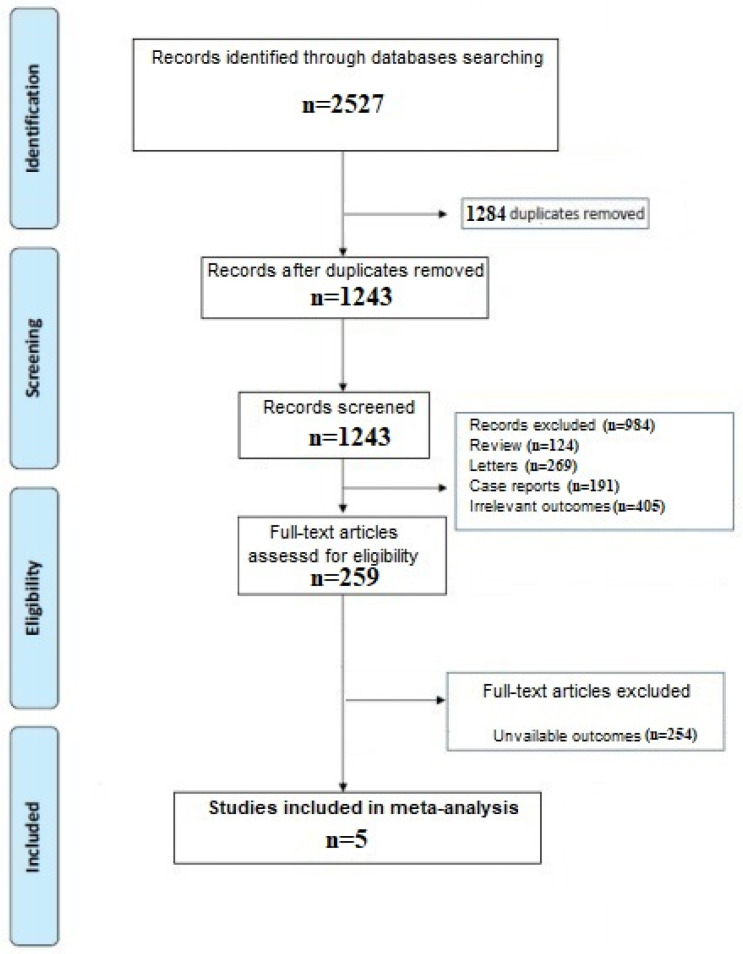
PRISMA flow diagram.

A total of 2527 articles were obtained by our search strategy. After excluding duplicates and preliminary screening, 1243 full-text articles were assessed for eligibility and 984 studies were excluded for not meeting the inclusion criteria while 254 for unavailable outcome, leaving 5 investigations fulfilling the inclusion criteria [6,7,16,17,18]. A flow diagram of the literature search and related screening process is shown in Figure 1.

### 3.2. Study Characteristics

Overall, 9623 COVID-19 patients [3707 males (38.5%)] were included in the analysis. The general characteristics of the included studies are summarized in Table 1. Despite that concomitant comorbidities were not systematically investigated by the analyzed studies, HT, DM and CKD were the most frequently observed [*n* = 1526/2692 (56.6%), *n* = 861/2629 (31.9%) and *n* = 824/2692 (30.6%)], respectively. Quality assessment showed that all the studies were of moderate-high quality according to the NOS scale. 

### 3.3. Pooled Prevalence of Anemia in COVID-19 Patients

**Figure 2:**
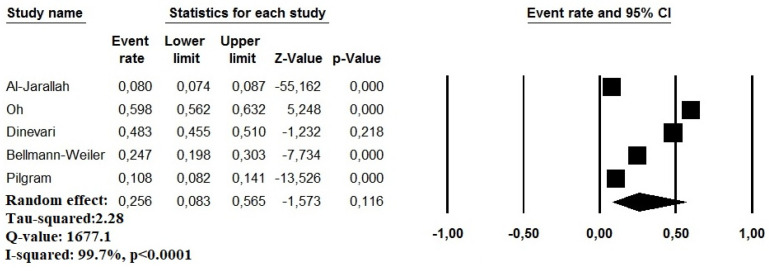
Pooled prevalence of anemia in COVID-19 patients.

For the primary outcome of the study, the prevalence of anemia among COVID-19 patients ranged between 8.0% and 59.8% [6,7,16,17,18]. A random effect model revealed a pooled prevalence of anemia in 25.6% of cases (95% CI: 8.3–56.5%). A high heterogeneity was observed in the analysis (I^2^ = 99.7%) (Figure 2). The relative funnel plot is presented in Supplementary File S2, Panel A.

### 3.4. Meta-Regression

Meta-regression analysis revealed a direct relationship between the prevalence of anemia and COVID-19 infection using age (Coeff. 0.091, 95% CI: 0.024 to 0.159, *p* = 0.007) and CKD (Coeff. 0.082, 95% CI: 0.019 to 0.131, *p* = 0.004) as moderating variables. Conversely, an inverse relationship was observed with male gender (Coeff. −0.189, 95% CI: −0.230 to −0.140, *p* < 0.0001). No associations were observed using HT (*p* = 0.68) and DM (*p* = 0.85) as moderators.

### 3.5. Anemia and Mortality Risk in COVID-19 Patients 

**Figure 3:**
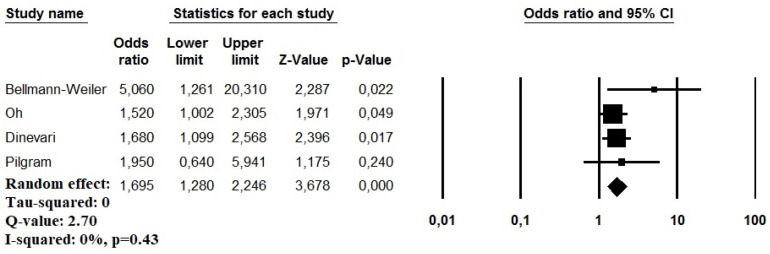
Forest plot investigating the mortality risk due to anemia in COVID-19 patients using a random-effect model.

Regarding the secondary outcome, four studies reported an aOR for the mortality risk in COVID-19 patients with anemia (*n* = 2692 patients, 1468 males) [7,8,17,18]. The variables used by each study to determine the aOR for the short-term mortality risk are presented in Table 2. On pooled analysis, patients with anemia showed a significant higher mortality risk in the short-term period (OR: 1.69, 95% CI: 1.28–2.24, *p* < 0.001, I^2^ = 0%) (Figure 3). The visual inspection of the relative funnel plot did not reveal significant evidence of publication bias (Supplementary File S2, Panel B).

### 3.6. Sensitivity Analysis

To evaluate the robustness of the results association, we performed a leave-one-out sensitivity analysis by iteratively removing one study at a time and recalculating the summary OR. The summary ORs remained stable (ranging between OR: 1.88, 95% CI: 1.52–2.18, *p* < 0.001 and OR: 1.34, 95% CI: 1.12–1.46, *p* < 0.001), indicating that our results were not driven by any single study.

## 4. Discussion

The present analysis demonstrates that anemia represents a relative common comorbidity in COVID-19 patients, being present in about one out of four infected patients. This association, which seems to be more frequent in women, was directly influenced by aging and concomitant presence of CKD. More importantly, SARS-CoV-2 infected patients with anemia had an approximately 70% higher risk of death in the short-term period compared to those without. Our findings are in accordance with the results presented by Al-Jarallah et al. who reported that COVID-19 patients having a hemoglobin > 10 g/dL had lower odds of dying than those who were considered anemic (i.e., Hb < 10 g/dL) (aOR: 0.33, 95% CI: 0.20 to 0.55, *p* < 0.001) [16]. However, considering that these authors used in their multivariate analysis those patients without anemia as a reference group, we excluded this investigation from global risk estimation in our meta-analysis.

The high heterogeneity observed in the pooled prevalence analysis performed in our study is probably multifactorial. Indeed, both the limited number of studies satisfying the inclusion criteria as well as the relative few numbers of patients enrolled represent, per se, a potential source of heterogeneity. Besides, inherited biases derived from the original investigations may have further contributed to the heterogeneity level observed. In fact, different levels of methodological quality, sampling methods and definitions of anemia may have produced significant differences among studies. However, some part of the observed heterogeneity between studies could be explained by the results of the meta-regression. To this regard, it has been already reported that rates of anemia in men increased monotonically with age, while that of women increased bimodally with peaks in age groups 40–49 years and 80–85 years [19]. Furthermore, older COVID-19 patients have a higher risk of mortality, but in this case, women with anemia seem to have a worse outcome compared to males who generally have been reported to be at higher risk of death [20]. This difference can be explained by considering that some research has shown that the risk for CKD is slightly greater in women than in men [21] and that prominent gender disparities in CKD prevalence do exist among different countries [22]. However, the absence of heterogeneity in the estimation of mortality risk in COVID-19 patients with anemia as well as the results of the sensitivity analysis confirmed the robustness of our results. 

From a pathophysiological perspective, Hb concentration represents one of the most important markers of oxygen-carrying capacity in the bloodstream. Therefore, anemia can further reduce oxygen delivery to peripheral tissue in COVID-19 patients who have an increased oxygen demand due the interstitial pneumonia [7,23,24]. Unfortunately, we cannot assess the potential prognostic impact of different types of anemia and relative pathophysiological pathways, such as deficient, excessive peripheral destruction, hereditary or blood loss. To this regard, adequate studies are needed to elucidate this issue. Probably, a major contributing role could have been played by the impairment of iron metabolism due to the underlying infection, resulting in the reduced availability of the metal for erythropoiesis and the production of Hb [8]. Our findings may have important implications in daily clinical practice: the prompt identification of patients with anemia remains critical to identify vulnerable populations who would require prioritization in treatment and close monitoring if infected, also in out-of-hospital settings.

### Limitations

Our study has several limitations related to both the design of the reviews studied with all inherited biases and the relatively few investigations conducted on the issue. Indeed, only a few studies have analyzed the relationship between anemia and mortality risk, limiting our results and conclusions. Moreover, the relatively high heterogeneity observed in the estimation of the pooled prevalence of anemia in SARS-CoV-2 patients, which probably depends on the inclusion criteria as well as the design of the studies and different anemia criteria, may have led to infirm conclusions. However, the sensitivity analysis performed partially mitigates these limitations, confirming the validity of our results. Doubtless, anemia is not itself a disease but generally the manifestation of other underlying pathological conditions, often influenced by different concomitant comorbidities and/or risk factors. Unfortunately, we were not able to assess the causes of anemia in the analysis since none of the revised manuscripts provided such information. Further larger clinical studies are needed to confirm our preliminary results, also comparing the prognostic impact of different types of anemia in COVID-19 patients.

## 5. Conclusions

Anemia is present in about 25% of patients with COVID-19 infection and is associated with an increased risk of short-term mortality. The present results further reinforce the concept that concomitant comorbidities remain important predictors of COVID-19 patient outcome.

## Figures and Tables

**Table 1:** General characteristics of the population enrolled. IQR: Interquartile range; R: Retrospective study; P: Prospective study; S: Single center study; Multi: Multicenter study; NS: Non survivors; HT: Arterial Hypertension; HF: Heart failure; CAD: Coronary artery disease; COPD: Chronic obstructive pulmonary disease; CKD: Chronic kidney disease; Hb: Haemoglobin; NOS: Newcastle–Ottawa quality assessment scale; NR: Not reported.

Author	Type	N° of pts	Mean Age, Years [IQR]	Males, N (%)	Anemia Patients (%)	NS, *n* (%)	HT, N (%)	DM, N (%)	Cancer, N (%)	HF, N (%)	CAD, N (%)	COPD, N (%)	CKD, N (%)	Anemia Cut-Off (Hb g/dL)	NOS
Al-Jarallah, et al. [16]	R Multi	6931	44.1 ± 17.2	2221 (66.1)	554 (7.9)	176 (2.5)	NR	NR	NR	NR	NR	NR	NR	Hb 10 g/dL	6
Oh, et al. [7]	R Single	733	65.0 ± 16.0	372 (50.8)	438 (59.8)	219 (29.9)	559 (76.3)	357 (48.7)	145 (19.8)	117 (16.0)	NR	88 (12.0)	271 (37.0)	Hb 12 for ♀ Hb 13 for ♂	8
Dinevari, et al. [17]	P Single	1274	64.3 ± 17.1	706 (55.4)	615 (48.2)	481 (37.7)	504 (39.5)	288 (22.6)	56 (4.6)	NR	NR	NR	112 (8.7)	Hb 12 for ♀ Hb 13 for ♂	7
Bellmann-Weiler, et al. [18]	R Multi	259	68 (53–80)	157	64 (24.7)	32 (12.3)	124 (47.8)	45 (17.3)	21 (8.1)	9 (3.4)	35 (13.5)	23 (8.8)	15 (5.7)	Hb 12 for ♀ Hb 13 for ♂	8
Pilgram, et al. [6]	R Multi	426	NR	251 (58.9)	46 (38.0)	12 (2.8)	339 (80.7)	171 (41.1)	85 (20.7)	132 (33.1)	133 (33.5)	53 (12.7)	426 (100)	Hb 10 g/dL	7

**Table 2:** Adjustment variables used to determine the adjusted OR for anemia and the risk of mortality. Oxygen saturation in arterial blood; LDH: Lactate dehydrogenase; CRP: C-reactive protein; CVD: Cardiovascular disease; eGFR: estimated glomerular filtration rate; PCT: Procalcitonin, HLD: Hyperlipidaemia; CKD: Chronic kidney disease.

Author	
Oh, et al. [7]	Age > 65; Male sex, DM, HT, HLD, Heart disease, CKD, Platelet, Creatinine
Dinevari, et al. [17]	Age, Hypoxia, Respiratory disease, Diabetes, Smoking, Disease severity
Bellmann-Weiler, et al. [18]	Age, CVD, COPD, eGFR, Leukocytes, PCT
Pilgram, et al. [6]	Age, HF, AF, Cerebrovascular disease, immunosuppressive medication, SO2, Dyspnea, LDH, Lymphocytes, Platelets, CRP.

## Data Availability

All the data pertaining to the study are available in the manuscript.

## Supplementary Materials

The following are available online at https://www.mdpi.com/article/10.3390/idr13040085/s1. Supplementary File S1. PRISMA Checklist. Supplementary File S2. Funnel plots for (A) the pooled prevalence of anemia in COVID-19 patients and (B) for the mortality risk due to anemia in COVID-19 patients.
