# Supplementary figures and images for: Prognostic Role of Anemia in COVID-19 Patients: A Meta-Analysis
Source: Infect Dis Rep. 2021 Oct 31;13(4):930–7. doi: 10.3390/idr13040085 (PMC8628963; doi:10.3390/idr13040085)

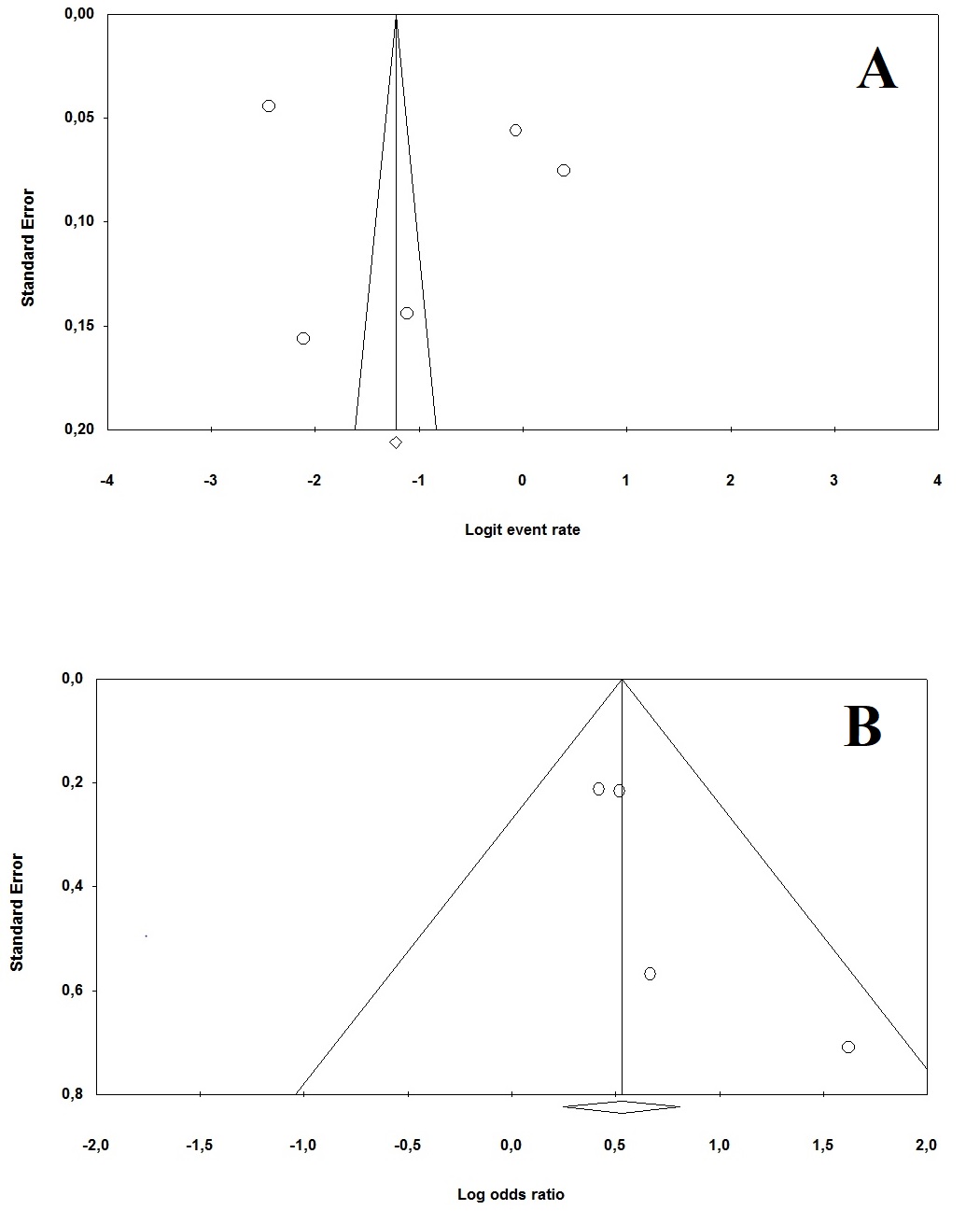

Supplement: Supplementary file 1 [file idr-13-00085-s001.zip › Supplementary file S2.jpg]
